# Nurse Led Extubation in Adult PACU - A Lean Process

**DOI:** 10.12669/pjms.38.1.5337

**Published:** 2022

**Authors:** Zulfiqar Memon, Andrew Gladney, Jubil Thomas, Shankar Lal

**Affiliations:** 1 Dr. Zulfiqar Memon, Consultant Anaesthetist, Our Lady of Lourdes Hospital, Drogheda, Republic of Ireland; 2 Andrew Gladney, CNM 3, Peri-operative Division, Our Lady of Lourdes Hospital, Drogheda, Republic of Ireland; 3 Dr. Jubil Thomas, Consultant Anaesthetist, Our Lady of Lourdes Hospital, Drogheda, Republic of Ireland; 4 Dr. Shankar Lal, Trainee Anaesthetist, Our Lady of Lourdes Hospital, Drogheda, Republic of Ireland

Operating room productivity has always been an area of focus to enhance access for patients and the financial solvency. Usually it takes minimum of 30 minutes between each case when patients are extubated in theater and cleaning process before the next patient. There are several studies published regarding the role, efficacy and complications of nurse led extubation in intensive care and in post-operating care unit (PACU). These studies revealed several factors responsible for the potential airway complications associated with Nurse Led Extubation but the overall incidence of complications was similar to the Anaesthetist Led Extubation.^1^ Nurse led extubation of patients in ICU is a norm in Ireland. We introduced Nurse Led Extubation of adult patient in PACU in our hospital. We drafted procedural policy following Lean six sigma process for health care management and quality improvement for the same.

Lean is a set of tools used with principles of six sigma to improve the operating theater efficacy.^2^ The anaesthesia consultants based teachings and hands-on were organised for qualified PACU nurses. The nurses who successfully completed the course of airway teaching and hands-on, were assessed with clinical case based scenario competency tests where the level of knowledge and problem solving were graded as Poor, Fair, Good, and Excellent. Nurses declared successful in competency tests were allowed to perform patient’s tracheal extubation in PACU.

The training leadership included consultant anaesthetists, nursing chief of operating rooms and chief of nursing staff. Initial extubations were supervised by consultant anaesthetists. Further extubations were supervised by chief recovery nurse of the day, trained and experienced at extubations. At all times anaesthesia consultants and senior anaesthesia NCHDs remained immediately available for any help.

To the best of our knowledge our hospital is the pioneer in introducing the policy and guidelines on Nurse Led Extubation in adult PACU.

## Authors’ Contribution:

**ZM and JT:** Drafted policy on Nurse LED Extubation, Nurse Training, and Final Write-up.

**AG:** Co-Author for Policy making, Data collection and Nurse Training.

**SL:** Data Collection, Initial write-up.
